# Advances in Proteomic Technologies and Its Contribution to the Field of Cancer

**DOI:** 10.1155/2014/238045

**Published:** 2014-09-07

**Authors:** Mehdi Mesri

**Affiliations:** Office of Cancer Clinical Proteomics Research, National Cancer Institute, NIH, Bethesda, MD 20892, USA

## Abstract

Systematic studies of the cancer genome have generated a wealth of knowledge in recent years. These studies have uncovered a number of new cancer genes not previously known to be causal targets in cancer. Genetic markers can be used to determine predisposition to tumor development, but molecularly targeted treatment strategies are not widely available for most cancers. Precision care plans still must be developed by understanding and implementing basic science research into clinical treatment. Proteomics is continuing to make major strides in the discovery of fundamental biological processes as well as more recent transition into an assay platform capable of measuring hundreds of proteins in any biological system. As such, proteomics can translate basic science discoveries into the clinical practice of precision medicine. The proteomic field has progressed at a fast rate over the past five years in technology, breadth and depth of applications in all areas of the bioscience. Some of the previously experimental technical approaches are considered the gold standard today, and the community is now trying to come to terms with the volume and complexity of the data generated. Here I describe contribution of proteomics in general and biological mass spectrometry in particular to cancer research, as well as related major technical and conceptual developments in the field.

## 1. Introduction

Although remarkable advances in cancer research have extended our understanding of how cancer develops, grows, and metastasizes, it is projected that close to 600,000 Americans will die from one of more than 200 types of cancer in 2013. Moreover, because an excess of 75 percent of cancer diagnoses occur in those aged 55 and older and this segment of the population is increasing in size, the number of cancer-related deaths will increase dramatically in the future. As a result, cancer is projected to soon become the number one disease-related killer of Americans. This trend is also observed globally, and it is estimated that, in 2030, more than 13 million people worldwide will die of cancer [1]. While significant amounts of resources are devoted to cancer research, the complexity and multifaceted nature of cancers reflect the obstacles to unravel the etiology of cancer and control and ultimately cure this debilitating disease. The heterogeneity and complexity of cancer progression originate from the complex interplay of genomic aberrations and immunological, hormonal, environmental, and other factors, acting individually or in concert which constitute the hallmarks of cancer.

The proteome is the operating machinery for nearly all biological functions; its abundance and interactions are precisely controlled and it is the link between the genome and phenotypes. Proteins can be present at vastly different abundances, expressed in various sizes, shapes, and charges, and have more complex twenty amino acid forms in contrast to the four nucleotides of the genome itself. It undergoes dynamic changes in different cells, tissues, and organs during development, in response to environmental stimuli and in disease processes. Understanding the dynamics of protein interactions with other proteins, nucleic acids, and metabolites is the key to delineating biological mechanisms and understanding disease including cancer. Genomic sequencing has been the focus of attention in recent decades and has produced a wealth of information. However, proteins are the component that functionally governs cellular processes. Moreover, variation in levels of DNA or transcripts does not correlate well with protein abundance [2]. Thus, proteomics bridges the gap between genomic information and functional proteins and translates this information. The possibility to systematically quantify protein abundances positions proteomics to monitor heterogeneous alterations in multiple pathways and mechanisms that drive the transformation of the malignant phenotype. Proteomics can be considered as an integral part of cancer research to identify biomarkers to detect patients at the early stage, monitor drug response of tumors, understand mechanisms that lead to cancer pathogenesis, and design new therapeutics. Scientists and oncologists thus use the various proteomics tools, design experiments, and interpret results of proteomics to determine the causative mechanisms, guide prognostication, and even develop precision medicine for cancer treatment.

## 2. Genomics

A key role of proteins in realizing the full potential of the human genome project (HGP) is linking the genome to normal and disease phenotypes. The HGP has changed many aspects of human biology and medical research including cancer. Despite many skeptics, the HGP became a reality by daring goals and new technology platforms [3]. Advanced technology platforms for sequencing dramatically changed the study of genes and gene regulation in all organisms [4]. The HGP has made all genes accessible to biologists by providing a part list of genes and putative protein products and stimulated a new perspective in studying biological processes through systems biology. Furthermore, the HGP has helped the creation of whole new commercial sector with high throughput instruments, reagents, and services and opened the door for large data sets, open-source data along with large-scale application of bioinformatics. The field has transformed our thinking about cancer diagnostics and targeted therapies. Despite all of these achievements skeptics are still concerned about the slow progress in transforming public health.

Both endogenous and exogenous stimuli may result in evading the normal regulatory mechanisms of the cells with ultimate aberrant phenotypes enabling cancer cell to proliferate, invade, and metastasize. Tumors contain endogenous aberrations that are largely caused by somatic alterations of genome that result in mutations of oncogenes and/or tumor suppressors [5]. For example, deletions, insertion, copy number variations, and mutations can drive initiation and progression of cancer [6]. Genomics, the comprehensive large scale analysis of gene expression in biological specimens to determine their associations with disease or treatment, has particularly been growing since 2005. New types of sequencing instruments that permit amazing acceleration of data-collection rates for DNA sequencing were introduced by commercial manufacturers including platforms such as massively parallel sequencing (MPS). For example, single instruments can generate data to decipher an entire human genome within only 2 weeks. It is anticipated that instruments that will further accelerate this whole genome sequencing data production timeline to days or hours will be a reality in the near future [7].

The main challenge in genomics today is to understand the role of molecular aberrations in various diseases such as cancer [8]. The Cancer Genome Atlas (TCGA) project (http://cancergenome.nih.gov/) and the International Cancer Genome Consortium (http://www.icgc.org/) aim to determine the genomic aberrations in human cancer types and their roles in pathophysiology of cancers. TCGA launched as a three-year pilot in 2006 by the National Cancer Institute (NCI) and National Human Genome Research Institute (NHGRI) in the United States. TCGA pilot project confirmed that an atlas of changes could be created. It also proved that a network of research and technology teams could pool the results of their efforts and develop an infrastructure for making the data publicly accessible. Moreover, it proved that public availability of data would enable researchers to validate important discoveries. The success of the pilot led the National Institutes of Health (NIH) to commit major resources to TCGA to collect and characterize more than 20 additional tumor types (http://cancergenome.nih.gov/). The ultimate goal is to translate genomics data into clinical applications such as routine clinical screening and precision medicine.

Classification of cancer and diagnosis has been based on cellular morphology and histological architecture. However, patients with similar histopathology, cancer staging, and treatments have shown variable clinical outcomes. Such methods are subjective and prone to interpretation variability, and pathologists do not always agree on the diagnosis. Therefore, diagnostic tools for cancers have evolved from histology to methods such as genomic testing with FISH, microarrays, and chromosome karyotype analysis. For example, gene expression profiles can be used as unique molecular signatures to aid diagnosis and classify histopathologically similar tumors into biologically distinct subtypes [9]. Molecular signatures can be used to identify patients with high risk for occurrence and poor survival and receive targeted therapies. Mammaprint is a Food and Drug Administration (FDA) approved breast cancer recurrence microarray-based test [10]. The Oncotype DX assay is used to predict risk for distant recurrence among invasive breast cancers [11]. A recent example of genomic signature for prognosis prediction of stages II and III colorectal cancer is ColoPrint [12]. In addition to biomarkers, genomics has led to discovery of new genes not previously known to play a causal role in cancer and leads to cancer therapeutics [3]. Some of the first sequencing attempts were focused in receptor tyrosine kinases (RTKs) in early 2000s. The studies indicated mutations in BRAF in 50% of melanomas [13], PIK3CA in 25%–30% of breast and colorectal cancers [14], and EFGR in 10%–15% of non-small cell lung cancers [15–17]. Interestingly some of the findings have resulted in a major impact on drug development and clinical treatment, including the development of selective RAF and MEK inhibitors that have produced dramatic remissions in melanoma and the ability to target the use of EGFR inhibitors to the subset of lung cancer patients who derive benefit.

## 3. Proteomic Challenge

By analogy, democratization of protein studies by generating a broader and deeper parts list to enable systems biology as well as deciphering protein interactions and biological signaling that mediate physiological and pathological conditions can have a major impact on medicine parallel to genome project [4]. This can take the form of cataloging all of the components, functionalization of the individual proteins, and putting the parts into relevant networks and circuitries and learning how these networks collectively process and execute their activities. The genome project identified all the genes and by inference all proteins. The challenge now is how these entities are integrated into molecular mechanisms resulting in phenotypes. An integral component of this system approach is requirement for technologies that can systematically identify and quantify all proteins, protein isoforms, and protein interactions. Proteins can take many different forms, for example, posttranslational modifications (PTMs), single-nucleotide polymorphisms (SNPs), and alternative splicing of proteins, resulting in numerous different structures of individual primary products, making the dimensions of proteome much larger than the human genome. Genomics alone cannot inform and delineate this protein diversity and its functional consequences.

**Figure 1:**
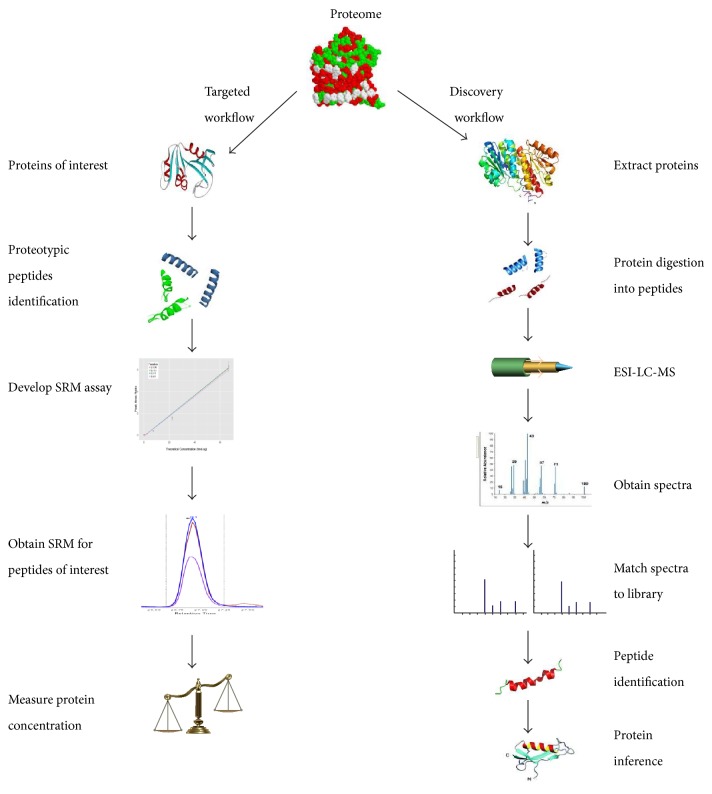
Discovery-based versus targeted proteomics workflows using mass spectrometry.

Mass spectrometry platforms are the workhorse of experimental proteomics for protein analyses and in the last five years there has been significant progress in sensitivity, throughput, type, and depth of proteome analysis. The goals of innovation are concentrated on increasing signal/noise in identifying and sequencing peptides, detecting and quantifying specific peptides with PTMs, SNPs, or splicing, and enhancing the throughput to make assays useful for clinical and population studies. Another area is to be able to study the dynamics of how proteomes change (in concentration and in structure) in response to exogenous signals and disease. There are two main proteomic platforms: discovery-based versus targeted proteomics workflows. Discovery-based proteomics, which detect a mixture of hundreds to thousands of proteins, has been the standard approach in research to profile protein content in a biological sample which could lead to the discovery of protein candidates with diagnostic, prognostic, and therapeutic values. In practice, this approach requires significant resources and time and does not necessarily represent the goal of the researcher who would rather study a subset of such discovered proteins under different biological conditions. In this context, targeted proteomics is playing an increasingly important role in the accurate measurement of protein targets in biological samples with the expectation of elucidating the molecular mechanism of cellular function via the understanding of intricate protein networks and pathways (Figure 1).

## 4. Advances in Discovery-Based Proteomics in Cancer

Proteomic technologies encompass a whole array of methods such as electrospray ionization-liquid chromatography tandem mass spectroscopy (ESI-LC-MS), matrix assisted laser desorption ionization time of flight (MALDI-TOF), surface enhanced laser desorption ionization time of flight (SELDI-TOF), MALDI MS imaging (MALDI-MSI), two-dimensional gel electrophoresis (2-DE), laser capture microdissection-MS (LCM-MS), and protein microarray, which can be used to derive important biological information to aid scientists and clinicians in understanding the dynamic biology of their system of interest such as in cancer [18–20].

### 4.1. ESI-LC-MS

ESI-LC-MS is a technology which produces gaseous ions carrying analytes byapplication of an electric potential to a flowing liquid in thepresence of heat. This causes formation of a spray upon high voltage, causing droplets tobecome electrically charged. Droplets eventually become unstable andexplode into even finer droplets and subsequently cause desorption of the analyte ions, which are then passed to themass spectrometer [21]. Most types of mass detection systems can be used with ESI including TOFs, ion traps (ITs), and quadrupoles. This technology can adopt various forms such as label-free or isotope labeling (metabolic labeling, ^18^O labeling), and chemical labeling (isotope coded affinity tag (ICAT), isobaric tags (iTRAQ), tandem mass tag (TMT), and dimethyl labeling).

#### 4.1.1. Label-Free

Quantitation method is relatively inexpensive and much easier to perform. This method uses spectral counting by measuring the frequency with which the peptide of interest has been sequenced by the MS, that is, the number of spectra for each peptide or protein being proportional to the amount of protein in the sample. This method can be used for biomarker discovery which normally requires high sample throughput by comparing peak intensity from multiple LC-MS data [22, 23]. Many studies have used label-free proteomics in cancer such as comparative proteomic analysis of non-small cell lung cancer [24] and proteins associated with metastasis in paraffin-embedded archival melanomas [25]. Application of this method for the analysis of changes in protein abundances in complex biological samples has certain limitations. For instance measuring small changes in the quantity of low-abundance proteins, which is often masked by sampling error, can be difficult. However, the method has an excellent linear dynamic range of about three orders of magnitude. Additionally, run to run analysis of the samples can exhibit differences in the peak intensities of the peptides as a result of sample processing requiring normalization. Additional issues may include experimental drifts in retention time and* m*/*z* (mass-to-charge ratio) complicating accurate comparison of multiple LC-MS data sets, chromatographic shifts as a result of multiple sample injections onto the same reversed-phase HPLC column, and unaligned peak comparison resulting in large variability and inaccuracy in quantitation. Also, large volume of data acquisition during LC-MS/MS requires the data analysis of these spectra to be automated. Computer algorithms have been developed to solve these issues and automatically compare the peak intensity data between LC-MS samples at a comprehensive scale [26, 27].

#### 4.1.2. Isotopic Labeling

Isotopic labeling can be either* in vivo* or* in vitro* by incorporating stable isotope into proteins or peptides for comparative analysis. Labeling techniques allow for multiplexing several samples to be analyzed and mitigate experimental variability inherent in sample processing.

- * Metabolic Labeling.* In this* in vivo* method cells are cultured with media containing isotopically labeled amino acids (13C and 15N) which are incorporated into the proteome during cell growth. In this method which requires metabolically active cells, samples grown with different labeled amino acids can be pooled for analysis [28]. More recently a stable isotope labeling with amino acids in cell culture (SILAC) mouse with a diet containing either the natural or the 13C6-substituted version of lysine was introduced [29]. With no effect on growth or behavior, MS analysis of incorporation levels allowed for the determination of incorporation rates of proteins from blood cells and organs. Mann's group has also introduced super-SILAC method by combining a mixture of five SILAC-labeled cell lines with human carcinoma tissue. This generated hundreds of thousands of isotopically labeled peptides in appropriate amounts to serve as internal standards for mass spectrometry-based analysis [30]. Super-SILAC can play a role in expanding the use of relative proteomic quantitation methods to further enhance our understanding of cancer biology and a tool for biomarker discovery [31]. Some of the disadvantages of metabolic labeling include incomplete labeling in cell culture medium which will affect accurate relative quantitation, labeling of all proteins necessitating purification, metabolic lability of amino acid precursors, and protein turnover [32].
- * Proteolytic* ^*18*^ *O Labeling.* In this technique ^18^O is incorporated during proteolytic digestion [33]. Differential ^16^O/^18^O coding relies on the ^18^O exchange where two ^16^O atoms are typically replaced by two ^18^O atoms by enzyme-catalyzed oxygen-exchange in the presence of H_2_^18^O. The resulting 2–4 Da mass shift between differentially labeled peptide ions permits identification, characterization, and quantitation of proteins from which the peptides are proteolytically generated [34]. ^18^O labeling bears at least two potential shortcomings: inhomogeneous ^18^O incorporation and inability to compare multiple samples within a single experiment. Unlike chemical labeling method such as ICAT, ^18^O labeling is simple with limited sample manipulations. It is much cheaper than ICAT and SILAC, comparing the price of reagents needed to label proteins. SILAC may be the method of choice for labeling of cultured cells, while ^18^O labeling may be used for samples with limited availability such as human tissue specimens [34]. In contrast to ICAT, ^18^O labeling does not favor peptides containing certain amino acids (e.g., cysteine) nor does it require an additional affinity step to enrich these peptides. Unlike iTRAQ,^18^O labeling does not require a specific MS platform nor does it depend on fragmentation spectra (MS^2^) for quantitative peptide measurements. It is amenable to the labeling of human specimens (e.g., plasma, serum, and tissues), which represents a limitation of metabolic labeling approaches (e.g., SILAC). Taken together, recent advancements in the homogeneity of ^18^O incorporation, improvements made on algorithms employed for calculating ^16^O/^18^O ratios, and the inherent simplicity of this technique make this method a reasonable choice for proteomic profiling of human specimens (e.g., plasma, serum, and tissues) in the field of biomarker discovery.
- *Chemical Labeling.* At least three methods of chemical labeling have been in use: ICAT, TMT, and iTRAQ. The ICAT reagent consists of three elements: an affinity tag (biotin), which is used to isolate ICAT-labeled peptides; a linker that can incorporate stable isotopes; and a reactive group with specificity toward thiol groups (cysteines). The reagent exists in two forms, heavy (contains eight deuteriums) and light (contains no deuteriums), leading to a difference in molecular weight of 8 Da between the two different forms of the tag. For quantifying differential protein expression, the protein mixtures are combined and proteolyzed to peptides and ICAT-labeled peptides are isolated utilizing the biotin tag. These peptides are separated by liquid chromatography. The pair of light and heavy ICAT-labeled peptides co-elute, and the 8 Da mass difference is measured in a scanning mass spectrometer. The ratios of the original amounts of proteins from the two cell states are strictly maintained in the peptide fragments. The relative quantification is determined by the ratio of the peptide pairs. The protein is identified by computer-searching the recorded sequence information against large protein databases [35–37]. The main disadvantage of ICAT labeling technique is that it only binds to cysteine residues, which constitutes approximately 1% of the protein composition. Similar to ^18^O labeling, ICAT has the limitation of having only two labels available, resulting in frequent experimentation and high cost if multiple samples need to be compared.
- Multiplexed sets of reagents for quantitative protein analysis have been developed which enable comparing of a larger number of treatments including the development of the 4- or 8-plex isobaric tag (iTRAQ) [38] and the 2- or 6-plex TMT [39] labeling techniques. The former can compare up to eight and the latter up to six samples in a single analysis. In these methods, both N-termini and lysine peptides are labeled with different isobaric mass reagents such that all derivative peptides are isobaric and indistinguishable. The different mass tags can only be distinguished upon peptide fragmentation. As each tag adds an identical mass to a given peptide, each peptide produces only a single peak during liquid chromatography and therefore only a single* m/z* will be isolated for fragmentation. The different mass tags only separate upon fragmentation, when reporter ions that are typical for each of the different labels are generated. The intensity ratio of the different reporter ions is used as a quantitative readout. One drawback of these methods is that only a single fragmentation spectrum per peptide may be available, while in quantitation-based MS1 scan, multiple data points are sampled resulting in a lower overall sensitivity. Some additional disadvantages of these techniques are the inconsistencies in labeling efficiencies and the high cost of the reagents. Use of standard operating protocols (SOPs) is recommended to achieve reproducible and reliable results with iTRAQ and therefore alleviating potential variability as a consequence of multistep sample preparations [40]. iTRAQ may also have limitations in dynamic range; experiments typically report fold changes of less than 2 orders of magnitude. From a purely technical point of view, this may be perceived as a limitation of iTRAQ for quantitative proteomics [41–43].

It is clear that both labeled and unlabeled MS analyses will continue to have their uses. Stable isotope labeling provides higher quality data at the analysis end. And with labeling methods, such as iTRAQ, the labels are introduced so late in the process that the experiment can be performed much faster than in earlier labeling methods. Even so, it is a lot more challenging technically than label-free techniques and also prone to systematic errors. The choice of isotope labeling technique is highly dependent upon experimental design, the scope of a particular analysis, and the sample or system being analyzed.

At the technology level, there is still room for progress. Performance in proteomics can be characterized by three factors: ion injection efficiency, cycling speed, and detector sensitivity. While the detectors are sensitive already at the level of detecting a single ion, the process of funneling the ions to the detector can be improved. Despite improvements in electrospray ionization injection, the majority of ions are still lost on their way to the detector. In addition, irrelevant molecules cannot be filtered out which result in generating a lot of noise. Improvements in these areas can enhance the signal/noise ratio. Furthermore, speeding up the cycling rate (the number of spectra per second) can increase the measurement depth. Given the wide dynamic range, this can in turn result in quantifying maximum proteins present in the sample [44].

### 4.2. MALDI-TOF

MALDI-TOF is a MS platform in which time of flight mass analyzer is usually coupled with MALDI. Ions are accelerated by an electric field followed by ion separation according to their* m*/*z* ratios by measuring the time it takes for ions to travel through flight tube. Specifically, the* m*/*z* ratio of an ion is proportional to the square of its drift time with heavier ions taking longer to travel. The sample for MALDI is uniformly mixed in a large quantity of matrix. The matrix absorbs the ultraviolet light and converts it to heat energy. A small part of the matrix heats rapidly and is vaporized, together with the sample [45]. MALDI-TOF-MS has become a widespread and versatile method to analyze a range of macromolecules in a wide range of samples. Its ability to desorb high-molecular-weight molecules and its high accuracy and sensitivity, combined with its wide mass range (1–300 kDa), make MALDI-TOF-MS a method of choice for the clinical chemistry laboratory for the identification of biomolecules in complex samples, including peptides, proteins, oligosaccharides, and oligonucleotides [46, 47]. The first reports of MALDI-TOF-MS biochemical analysis were published in the late 1980s from Karas and Hillenkamp lab [45]. Although being relatively young compared to other analytical techniques using mass spectrometry, there has been an enormous increase in the publication of MALDI-TOF-MS methods and applications in the literature. While ESI can efficiently be interfaced with separation techniques enhancing its role in the life and health sciences, MALDI, however, has the advantage of producing singly charged ions of peptides and proteins, minimizing spectral complexity.

### 4.3. SELDI-TOF-MS

SELDI-TOF-MS technique was introduced in 1993 by Hutchens and Yip [48] and later commercialized by Ciphergen Biosystems in 1997. SELDI-TOF-MS is a variation of MALDI that uses a target modified to reach biochemical affinity with the sample proteins. There are some differences between the two techniques. In MALDI, the sample is mixed with the matrix molecule in solution, and a small amount of the mixture is deposited on a surface to dry. This makes the sample and matrix cocrystallized after the solvent evaporated. On the other hand, in SELDI, the mixture is spotted on a surface modified with a chemical functionality such as binding affinity. There are different types of chemicals and substances bound to the protein arrays, including antibodies, receptors, ligands, nucleic acids, carbohydrates, or chromatographic surfaces (i.e., cationic, anionic, hydrophobic, or hydrophilic). Still wet, some proteins in the samples would bind to the modified surface, while the others will be washed off. Then the matrix is applied to the surface for crystallization with the sample peptides. In the binding and washing off steps the surface-bound proteins are left for analyses. Samples spotted on an SELDI surface are analyzed with TOF mass spectrometry (TOF-MS) [49, 50]. The strength of this technology is the integration of on-chip selective capture, relative quantitation, and partial characterization of proteins and peptides. The differential expression data obtained from this technology has been used for identification of biomarker candidates for various cancer types, such as prostate [51, 52], pancreas [53–55], lung [56–58], breast [59–61], melanoma [62], colon [63, 64], ovarian [65–67], and liver cancers [68, 69].

### 4.4. MALDI-MSI

MALDI-MSI is a powerful technique which allows investigating the distribution of proteins and biomolecules directly from a tissue section [70, 71]. This technique also permits investigation of the spatial and temporal distribution of biomolecules such as phospholipids without the need for extraction, purification, and separation procedures of tissue sections [72]. MALDI-MSI can help in molecular diagnosis on tissue directly in the environment of the tumors and can detect the tumor boundary or infiltration of adjacent normal tissue. It could also help to detect the early stage of pathology that presents no histological modifications and to prevent tumor recurrence at the site of surgical resection. One of the advances of MSI is the correlation of the MALDI images with histological information. MALDI-MSI software [73] superimposes the MALDI images over a macroscopic or microscopic optical image of the sample taken before MALDI measurement. MALDI-MSI has been used in clinical proteomics for biomarker discovery in a variety of diseases including cancer [74–78]. Development of SOPs and standardization of protocols for sample collection, storage, data acquisition, and enhancement of imaging resolutions and 3D tumor mapping are still needed to further improve its utility [79, 80]. Also the present levels of sensitivity allow the detection of a small group of cells but are not sufficient to detect discrete modification at a single cell level. A major advance for MALDI-MSI will be its coupling with positron emission tomography, X-ray, computed tomography instrumentation, and MRI for both preclinical and clinical research. The complementarities between noninvasive techniques and molecular data obtained from MALDI MS imaging will result in a more precise diagnosis [81]. Ultimately comparing the MRI image of a tumor and the image generated by MALDI-MSI at a molecular level will provide a comprehensive data set for diagnosis and treatment selection.

### 4.5. 2-DE

2-DE or two-dimensional gel electrophoresis technology was a pivotal turning point in the field of separation and has been shown to be a reliable and efficient method for separation of proteins based on mass and charge [82]. High resolution two-dimensional polyacrylamide gel electrophoresis (2D-PAGE) can resolve up to 10,000 protein spots per gel. This technique has been used in human tissue, plasma, and serum proteome analysis with or without prior fractionation [83–86]. Visualization of resolved proteins in the gel can be performed by staining methods such as Coomassie blue and silver staining [87, 88]. Some of the recent advances in silver staining products make it compatible with MS analysis too. To enable direct comparison of different mixtures of proteins, differential in-gel electrophoresis (DIGE) has been developed which permits simultaneous comparison of labeled proteins in different mixtures. In a typical experiment, two samples are labeled with different fluorescence dyes (Cy3 and Cy5) and mixed prior to electrophoresis and run in parallel with an internal standard labeled with a third dye (Cy2) for quantitative analysis [89, 90].

For identification purposes, gel-separated proteins can be digested into peptides. Analysis of the peptides can then provide a peptide mass fingerprint (PMF), which can be searched against theoretical fingerprints of sequences in protein databases. Alternatively, peptides can be sprayed into a tandem mass spectrometer (ESI) as they elute off a liquid chromatography (LC) column. The data can be searched for protein sequence and analyzed by the application of algorithms and comparison with theoretical production spectra of proteins in databases [91, 92].

2D-PAGE is a low throughput technology, labor intensive with low dynamic range, and prone to gel-to-gel variability. Although DIGE has shown improved accuracy, it is still a relatively low throughput method. It can be used in areas such as biomarker discovery, where high throughput processing of samples is not required [93].

### 4.6. LCM-MS

LCM-MS has proven an effective technique to harvest pure cell populations from tissue sections. Because proteome varies in different cells, the advent of laser capture microdissection has expanded the analytical capabilities of microproteomics by enabling protein analysis from extremely small samples. A typical protocol uses nanoscale liquid chromatography/tandem mass spectrometry (nano-LC-MS/MS) to simultaneously identify and quantify hundreds of proteins from LCMs of tissue sections from small tissue samples containing as few as 1000 cells. The LCM-dissected tissues are subjected to protein extraction, reduction, alkylation, and digestion, followed by injection into a nano-LC-MS/MS system for chromatographic separation and protein identification. The approach can be validated by secondary screening using immunological techniques such as immunohistochemistry or immunoblots [94].

LCM has significantly improved the analytical capabilities of comparative proteomic technologies to the extent that 2D-DIGE and quantitative gel-free mass spectrometry approaches have been coupled to LCM for proteomic analyses of distinct, pure cell populations [95–101]. The LCM technology allows for miniaturization of extraction and isolation and detection of hundreds of proteins (100–300 proteins) from different cell populations containing as few as 1000 cells. Additionally, it can detect and verify robust protein expression differences between different cell populations. Unlike traditional proteomic technologies, the LCM procedure requires as little as 1-2 *μ*g of protein. However, each step of the procedure requires greater care as the sample size decreases. Protein losses during extraction and separation become more significant as the protein detection limit (<0.75 *μ*g) is approached [94]. Other methods such as punch biopsy can be used to microdissect tissues for proteomic analyses [102], but because the three-dimensional view of boundaries of tissue structures is limited, punch biopsies can sample adjacent regions that are not of interest. In a recent paper published by Mueller et al. [103], the authors claimed that data derived from nonmicrodissected glioblastoma multiforme (GBM) can result in inaccurate correlations between genomic and proteomic data and subsequent false classifications. This is because molecular signals could be masked where sample tumor content is low or where the signal is strong in the stromal cells. Mueller et al. investigated 39 glioblastoma samples taken from tissue previously analyzed by the TCGA project. Using reverse phase protein array (RPPA) they measured the levels of 133 proteins and phosphoproteins, comparing LCM and non-LCM samples, finding differences in 44 percent of the analytes between the two types. They specifically investigated in more depth the genomic and proteomic data for epidermal growth factor receptor (EGFR) and phosphatase and tensin homolog (PTEN), two clinically important proteins in glioblastoma. While the researchers observed in both sample types increased EGFR protein and phosphoprotein levels in patients with increased EGFR gene copy number, they observed the increase in EGFR phosphorylation expected in carriers of EGFR mutations only in the LCM samples. In the case of PTEN, the researchers observed the expected decrease in PTEN levels in tumors with deep loss of PTEN or PTEN mutations only in the LCM samples. Additionally, they found the expected correlation between EGRF phosphorylation, PTEN levels, and phosphorylation of AKT only in the LCM samples which is regulated by the former two proteins. Mueller et al. also examined proteomic glioblastoma data previously generated by TCGA using non-LCM samples, again failing to observe the expected correlation between PTEN copy number or mutational status and PTEN protein levels. The authors recommend careful upfront cellular enrichment in biospecimens that form the basis for targeted therapy selection [103]. Further developments in LCM technology should facilitate effective sampling of specific cellular subtypes from tissue in a high throughput manner.

### 4.7. Protein Microarray

Protein microarray is a high throughput tool for studying the biochemical activities of proteins, tracking their interactions, and determining their function on a large scale [104]. Its main strength is that large numbers of proteins can be tracked in parallel. The chip usually consists of a support surface such as a glass slide, nitrocellulose membrane, bead, or microtiter plate, to which an array of capture proteins is bound. The commonest type of protein microarray may contain a large number of spots of either proteins or their ligands arranged in a predefined pattern, arrayed by robots onto coated glass slides, microplates, or membranes. The array may consist of antibodies to bind proteins of interest [105], enzymes that will interact with substrates, or substrates or ligands that will interact with applied proteins. Therefore, protein microarray formats can be divided into two major classes depending on what is immobilized on the support surface. In forward-phase protein array (FPPA) the capture antibody is first immobilized on a solid surface to capture the corresponding antigen in a test sample. The captured analyte is then directly detected with a fluorescent dye-conjugated detection antibody or detected indirectly with the detection antibody followed by a fluorescent dye-conjugated second antibody [106]. In this method, identification of a capture and a detection affinity reagent can be time-consuming. To bypass the requirement for two affinity reagents, reverse phase protein array (RPPA) may provide an alternative solution. In RPPA, test samples that could run into thousands are printed on the slide directly and detected with dye-conjugated antibodies [107]. RPPA assays are commonly used in tissue microarray and cell and tissue lysate microarray. While RPPA provides a high throughput platform, the specificity might be compromised to some degree owing to the use of single detection antibodies.

One technical downside to producing a reliable array is shelf-life. Most protein arrays use antibodies to deposit and be immobilized on the support surface which can denature the antibody and affect its recognition properties. Another bottleneck that chip manufacturers face is getting good quality and specific antibody against every protein in the human proteome which is a gigantic task. In addition to limited inventory of specific antibodies to PTMs (such as phosphorylation and glycosylation), generation of high throughput protein expression systems and purification including those with PTMs required for spotting the complete proteome under study is another challenge or may suffer from lack of reproducibility. Establishing standard criteria for array production and data normalization using noise models, variance estimation and differential expression analysis techniques would improve interpretation of microarray results [108].

The challenges in producing proteins to spot on the arrays fueled the development of a novel approach to protein microarrays technology called nucleic acid programmable protein array (NAPPA) which uses cell-free extracts to transcribe and translate cDNAs encoding target proteins directly onto glass slides. This approach eliminates the need to purify proteins, avoids protein stability problems during storage, and captures sufficient protein for functional studies [109, 110]. In recent studies NAPPA was coupled with MS and used for several applications, including the identification of peptide sequences for potential phosphorylation as well as a high throughput method for the detection of protein-protein interactions [111]. Moreover, the challenges of constructing solid-surface arrays holding thousands of proteins with different properties raised interest in protein-interaction assays in solution. Suspension-bead assays are particularly flexible and as a result suspension platforms were developed such as the Bio-Plex system from Bio-Rad Laboratories which uses Luminex's bead-based xMAP technology [112] as does the LiquiChip system from Qiagen Instruments. Suspension-bead arrays are flexible enough to tackle any sort of protein-ligand interaction by simply coupling the required proteins or ligands to different bead populations. Luminex beads, for example, enable simultaneous quantitation of up to 100 different biomolecules in a single microplate well. Rather than a flat surface, Bio-Plex assays make use of differentially detectable bead sets as a substrate capturing analytes in solution and employ fluorescent methods for detection [113].

## 5. Advances in Targeted-Based Proteomics in Cancer

**Figure 2:**
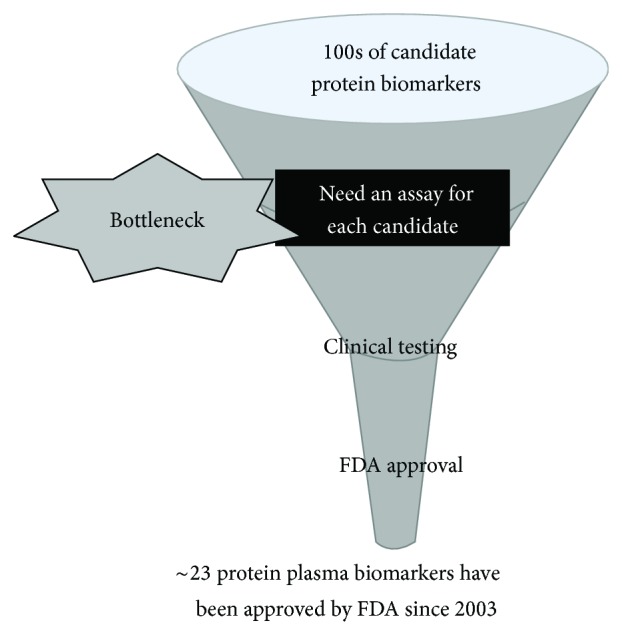
The assay bottleneck prevents potential protein diagnostics from becoming clinically useful.

**Figure 3:**
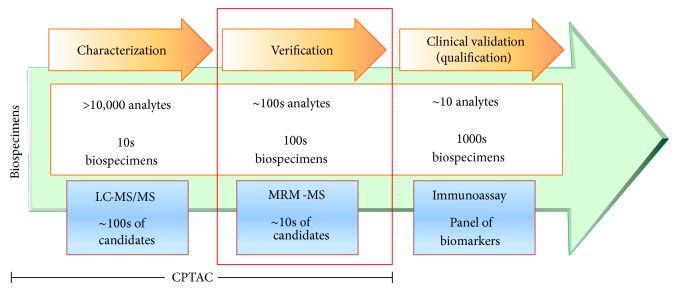
The incorporation of verification step into the NCI-CPTC pipeline bridging discovery and qualification.

The field of biomarkers, in particular, has benefited significantly from application of proteomic platforms over the last decade or more, with the goal of identifying simple noninvasive tests that can indicate cancer risk, allow early cancer detection, classify tumors so that the patient can receive the most appropriate therapy, and monitor disease progression, regression, and recurrence. A variety of biospecimens such as tissue, proximal fluids, and blood have been interrogated for protein or peptide markers identification. Thousands of publications have explored the potential use of individual proteins or collections of proteins as cancer biomarkers and have produced promising results [114, 115]. One study by Polanski and Anderson [116] has identified >1261 protein biomarker candidates for cancer alone. However, only 23 protein plasma biomarkers have cleared the US Food and Drug Administration (FDA) since 2003 as clinical biomarkers averaging <2 proteins per year over the last 12 years, while assays for at least 96 analytes have been developed and used as laboratory-developed tests (LDTs) [115]. Despite recent technical advances, there are still huge analytical challenges for clinically relevant identification of biomarkers in serum or plasma. This is compounded by the lack of analytical validation of a platform(s) for the precise and accurate measurements of identified analytes in a smaller set of clinical samples prior to proceeding to costly and time-consuming large-scale clinical trials (Figure 2). The Clinical Proteomic Technology Assessment for Cancer (CPTAC 1) of the NCI developed the innovative concept of biomarker “verification” which bridges discovery and validation. This pipeline has the potential to enable delivery of highly credentialed protein biomarker candidates for clinical validation (Figure 3). Targeted proteomic technologies such as multiplexed MS, protein arrays, and enzyme-linked immunosorbent assays (ELISAs) can fill this bridging space. Verification of candidates relies upon specific, multiplex quantitative assays optimized for selective detection of biomarker candidates and is increasingly viewed as a critical step in the protein biomarker development pipeline that bridges unbiased biomarker discovery to clinical qualification [117, 118].

### 5.1. Selected Reaction Monitoring-MS (SRM-MS) and Multiple Reaction Monitoring-MS (MRM-MS)

**Figure 4:**
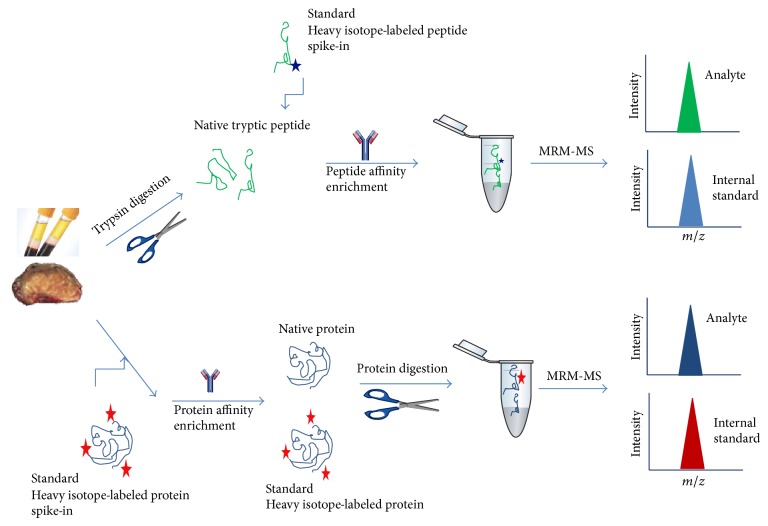
MRM-MS-based assay workflows (± immunoaffinity enrichment of proteins or peptides). SISCAPA workflow using proteolytic peptides as surrogates for their respective proteins, as illustrated in the top panel of the schematic, is a sensitive approach to measure protein concentrations using immunoaffinity enrichment of surrogate peptides prior to MRM-MS. To achieve quantitation of the targeted protein(s), they are digested to component peptides using an enzyme such as trypsin. A stable isotope standard (SIS, blue asterisk) is added to the sample at a known concentration for quantitative analysis. The selected peptides are then enriched using anti-peptide antibodies immobilized on a solid support. Following washing and elution from the anti-peptide antibody, the amount of surrogate peptide is measured relative to the stable isotope standard using targeted mass spectrometry. Alternatively, an assay can start with immunoaffinity enrichment of intact target proteins from biospecimens using an internal stable isotope-labeled protein standard (red asterisk, such as PSAQ approach) and an antibody, as illustrated in the bottom panel, followed by proteolysis and final quantitation of the target.

SRM-MS is a targeted technique that is completely different from the mass spectrometry approaches widely used in discovery proteomics. SRM is performed on specialized instruments that enable targeting of specific analyte peptides of interest and provides exquisite specificity and sensitivity [119–121]. SRM-MS is a nonscanning mass spectrometry technique, performed on triple quadrupole-like instruments (QQQ-MS) and in which collision-induced dissociation (CID) is used as a means to increase selectivity. In SRM experiments two mass analyzers are used as static mass filters, to monitor a particular fragment ion of a selected precursor ion. Unlike common MS-based proteomics, no mass spectra are recorded in a SRM analysis. Instead, the detector acts as counting device for the ions matching the selected transition thereby returning an intensity value over time. In MRM, multiple SRM transitions can be measured within the same experiment on the chromatographic time scale by alternating between the different precursor/fragment pairs. Typically, the triple quadrupole instrument cycles through a series of transitions and records the signal of each transition as a function of the elution time. The method allows for additional selectivity by monitoring the chromatographic coelution of multiple transitions for a given analyte [122, 123]. A schematic representation of MRM-MS-based assay workflows (± immunoaffinity enrichment of proteins or peptides) is depicted in Figure 4 and described in the following sections.

### 5.2. MRM-MS-Based Assay Development for Protein Verification

**Figure 5:**
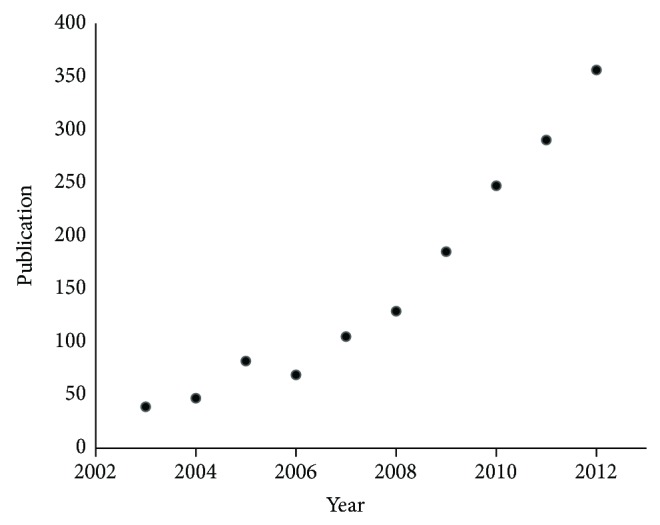
Increase in number of MRM publications in PubMed over the past decade.

MRM-MS method for quantification of biomolecules has been long in use (e.g., drug metabolites [124, 125], hormones [126], protein degradation products [127], and pesticides [128]) with great precision (CV < 5%) but has only recently been adopted for protein and peptide measurements. Stable isotope dilution (SID) multiple reaction monitoring MS (SID-MRM-MS) has emerged as one of the powerful targeted proteomic tools in the past few years. MRM mass spectrometry is being rapidly adopted by the biomedical research community as shown by increase in the number of publications in this area over the past decade (Figure 5). It has the advantage of accurately calculating protein concentrations in a multiplexed and high throughput manner, while avoiding many of the issues associated with antibody-based protein quantification [117]. SID-MRM-MS protein assays are based upon the quantitation of signature tryptic peptides as surrogates that uniquely represent the protein candidates of interest [129, 130]. To improve the specificity of the quantitative measurement for targeted analytes in MRM-based assays, a selection of three to five peptides per protein is selected [131]. Moreover, known quantities of synthetic stable isotope-labeled peptides (heavy peptides), corresponding to each endogenous peptide, are used as internal standard peptides (i.e., stable isotope-labeled internal standards or SIS). These SISs are identical to their endogenous analyte peptide counterparts with the exception of their masses (usually 6–10 Da more). For quantitation, specific fragment ion signals derived from the endogenous unlabeled peptides are compared to those from the spike-in SISs as ratios and are used to calculate the concentration of that protein [130, 131]. In SID-MRM-MS, the presence of SIS can calculate more accurate ratios with high sensitivity and across a wide dynamic range. The absence of an endogenous peptide signal typically means that the concentration of the peptide in the sample is below the detection limit of the instrument. Additionally the amount of SIS added should be optimized empirically in a preliminary study as this depends on the protein's individual relative abundance within a sample. High sensitivity and precision, combined with specific quantitation in a multiplex fashion, make MRM assays attractive for translational and clinical research [132, 133]. Targeted proteomics has also been recognized by the journal* Nature Methods* as the method of the year in 2012 [134].

There are many advantages to MRM-based assays which overcome major limitations of conventional protein assay technologies such as Western blot, IHC, and ELISA. Such advantages include moderate-to-high throughput capability, readily multiplexed assays, standards being readily synthesized, interferences being avoidable, use of internal standards for high interlaboratory reproducibility, and quantitation. Additionally, MRM assays have high molecular specificity, which does not require immunoassay-grade antibodies (those proteins for which no affinity reagent has been developed are accessible for routine quantification including isoforms and PTM analytes), and there is a large deployed instrument base. However, MRM assays are not easy to generate* de novo* and require expertise in addition to lack of validated reagents for most proteins [135, 136]. Over the past few years, the methods used to quantify proteins by MRM have steadily evolved and have been widely deployed. It has also been suggested recently that, considering the vast majority of protein identifications claimed from biological samples are still derived from Western blotting, it may be time that journal reviewers request that Western blotting results, or at least the assays that support these results, be validated by MS [136].

Recently in a landmark paper in* Nature Methods*, researchers have demonstrated the feasibility of both the development and application of MRM to reproducibly measure human proteins in breast cancer cell lysate across three labs in two countries in two continents. The international research collaboration, representing investigators from Fred Hutchinson Cancer Research Center (Seattle, Washington, USA), Broad Institute (Cambridge, Massachusetts, USA), and a team composed of researchers from the Korea Institute of Science and Technology and the Seoul National University College of Medicine (both Seoul, Republic of Korea), reported the development and application of 645 assays representing 319 proteins. The assays were deployed in multiplexed fashion in groups of at least 150 peptides to quantify proteins in a panel of breast cancer-related cell lines. Researchers were able to show that targeted mass spectrometry-based proteomic assay can be easily implemented anywhere, with minimal adjustments, while maintaining a high level of performance (accuracy, precision, and reproducibility), all essential for clinical implementation. Analyses of the results were able to recapitulate known molecular subtypes ascribed to breast cancer and also showed the added value of integrative analysis in identifying putative disease genes. This study demonstrates the tremendous promise on targeted proteomics to meet the interest of biologists and medical researchers and addresses the ability to replicate results from labs [137].

While adoption of targeted MS approaches such as MRM to study biological and biomedical questions is well underway in the proteomics community, there is no consensus on what criteria are acceptable and little understanding of the impact of variable criteria on the quality of the results generated. There is a wide range of criteria being applied to say that an assay has been successfully developed. Publications describing targeted MS assays for peptides frequently do not contain sufficient information for readers to establish confidence that the tests work as intended or to be able to apply the tests described in their own labs. To address these issues, a workshop was held recently at the NIH with representatives from the multiple communities developing and employing targeted MS assays. Participants discussed the analytical goals of their experiments and the experimental evidence needed to establish that the assays they develop work as intended and are achieving the required levels of performance. Using this fit-for-purpose approach, the group defined three tiers of assays distinguished by their performance and extent of analytical characterization. Participants also detailed the information that authors need to provide in their manuscripts to enable reviewers and readers to clearly understand what procedures were performed and to evaluate the reliability of the peptide or protein quantification measurements reported [138].

### 5.3. Enriching Analytes to Increase the Sensitivity of MRM-MS Assays

Many analytes require enrichment for MRM-based quantification of endogenous levels such as most proteins in plasma, regulatory and signaling proteins in cells and tissues, and PTMs. Many clinically relevant biomarkers, such as prostate-specific antigen (PSA) and the troponins (Tns), are expressed in low ng/mL level in plasma below the lower limit of detection of a QQQ-MS. There have been reports of improving sensitivities by abundant protein depletion strategy combined with minimal fractionation or instrument modification which could further improve the LOD/LOQ of MRM measurements. For instance, antibody-based depletion columns combined with minimal fractionation of tryptic peptides have been shown to improve sensitivity for proteins in plasma [131, 139, 140]. Furthermore, enhanced sensitivity for SRM-MS targeted proteomics through enhanced ion transmission efficiency using a dual stage electrodynamic ion funnel interface has been reported [141]. In another development, Fortin et al. used MRM cubed (MRM^3^), which enabled targeting protein biomarkers in the low nanogram/milliliter range in nondepleted human serum using a simple two-step workflow. This strategy takes advantage of the capability of a hybrid QQQ-MS/linear IT (LIT) mass spectrometer to further fragment the product ions monitored in Q3 [142].

### 5.4. PRISM

PRISM, reported very recently by Shi et al. [143], is an antibody-free strategy that involves high pressure, high resolution separations coupled with intelligent selection and multiplexing (PRISM) for sensitive selected reaction monitoring- (SRM-) based targeted protein quantification. The strategy uses high resolution reversed-phase liquid chromatographic separations for analyte enrichment, intelligent selection of target fractions via online SRM monitoring of internal standards, and fraction multiplexing before nanoliquid chromatography-SRM quantification [143]. This method has shown a major advance in the sensitivity of targeted protein quantification without the need for specific-affinity reagents. Applying this method to human plasma/serum demonstrated accurate and reproducible quantification of proteins at concentrations in the 50–100 pg/mL range. Excellent correlation between PRISM-SRM assay and those from clinical immunoassay for the prostate-specific antigen level was also noted [143]. A disadvantage of PRISM-SRM relative to SISCAPA (see Section 5.7) is reduced analytical throughput as a result of fractionation. However, even with limited fraction concatenation, moderate throughput (~50 sample analyses per week depending upon experimental details) can be achieved. For example, when quantifying a relatively large number of proteins (i.e., 100), all 96 fractions may contain target peptides; however, these fractions can still be carefully combined into 12 multiplexed fractions based on peptide elution times to achieve moderate throughput [143].

### 5.5. Parallel Reaction Monitoring (PRM)

PRM is a new targeted proteomics paradigm centered on the use of next generation, quadrupole-equipped high resolution and accurate mass instruments [144]. In PRM, made possible by the Q Exactive, the laborious development of SRM assays may be avoided. This instrument is similar to QQQ except that the third quadrupole is replaced with a high resolution, high mass accuracy Orbitrap mass analyzer. Whereas in SRM all transitions are monitored one at a time, the Q Exactive allows parallel detection of all transitions in a single analysis. Because all transitions can be monitored with PRM, one does not need to carry out laborious optimizations to generate idealized assays for selected transitions [145]. This will bring additional specificity because all potential product ions of a peptide, instead of just 3–5 transitions, are available to confirm the identity of the peptide [146] and that because PRM monitors all transitions, one need not have prior knowledge of, or preselect, target transitions before analysis. Also because many ions would be available, the presence of interfering ions in a full mass spectrum would be less problematic to overall spectral quality than interference in a narrow mass range [144]. In addition, the Q Exactive instrument is very flexible. Since one instrument can do both discovery and targeted analysis, this will allow researchers to use a discovery-based approach to identify a shortlist of interesting proteins and then use a targeted approach to follow those targets with high sensitivity under various conditions, all in a single experiment [145].

### 5.6. SWATH Acquisition

SWATH (sequential window acquisition of all theoretical mass spectra) acquisition is a novel technique that is based on data-independent acquisition (DIA) which aims to complement traditional discovery MS-based proteomics techniques and SRM methods [147]. In this strategy systematic queries of sample sets are made for the presence and quantity of any protein of interest. It consists of using the information available in fragment ion spectral libraries to mine the complete fragment ion maps generated using a data-independent acquisition method. In SWATH acquisition, the first quadrupole sequentially cycles 25 Da precursor isolation windows (swaths) across the mass range of interest and time-resolves fragment ion spectra for all the analytes detectable [147, 148] and therefore the potential to perform a significant larger number of SRM-like experiments concurrently. In SWATH-MS approach, the instrumental scanning speed has to be fast enough to allow acquiring an adequate number of data points across the typical chromatographic peak such that ion chromatography can be reconstructed with acceptable signal-to-noise ratio. SWATH acquisition, however, has a major drawback in that the data is incompatible with conventional database searching, and it seems a deconvolution algorithm to process the SWATH-MS data for database searching has not been achieved. There are a number of challenges in designing a deconvolution algorithm to process such complex data [148].

### 5.7. Protein Capture Enrichment

An alternative approach to immunoaffinity depletion (negative enrichment) and fractionation strategies is positive enrichment strategies which have also been extensively explored in proteomics for better detection of low-abundance peptides or proteins. These strategies include affinity enrichment of peptides or proteins and chemical enrichment of different subsets of the proteome including PTMs such as N-linked glycopeptides and phosphopeptides. Many of the enrichment strategies reported for general proteomics are also applicable to SRM applications.

Immunoaffinity capture of target proteins is probably the most effective method for sensitive detection of low-abundance proteins in complex samples [149, 150]. The immunoaffinity enrichment method coupled with MS has provided quantification of proteins in the low ng/mL range [151–153]. Nicol et al. [151] have demonstrated the immunoaffinity-SRM approach for the quantification of protein biomarkers for which ELISA assays are not available from lung cancer patients by using antibodies to enrich proteins, followed by digestion of captured proteins and subsequent SRM analysis. This approach enabled the quantification of multiple protein biomarkers in lung cancer and normal human sera in the low ng/mL range. In a different study, Kulasingam et al. reported the enrichment of endogenous PSA protein from 5 *μ*L of serum with a monoclonal antibody (mAb) followed by product ion monitoring using a linear ion-trap mass spectrometer [152] demonstrating quantification of PSA down to less than 1 ng/mL level with acceptable CVs. Recently, Niederkofler et al. [154] developed an assay that incorporates a novel sample preparation method for dissociating IGF1 from its binding proteins. The workflow also includes an immunoaffinity step using antibody-derivatized pipette tips, followed by elution, trypsin digestion, and LC-MS/MS separation and detection of the signature peptides in SRM mode. The resulting quantitative mass spectrometric immunoassay (MSIA) exhibited good linearity in the range of 1 to 1,500 ng/mL IGF1, intra- and interassay precision with CVs of less than 10%, and lowest limits of detection of 1 ng/mL [154]. Additionally, intact protein targets from samples, along with their recombinant heavy isotope-labeled internal protein standards, such as protein standard absolute quantification (PSAQ) approach [155–157] can be immunoprecipitated with antibodies prior to proteolysis and SID-MRM-MS. In 2004, Nelson et al. described an immunoaffinity-based MALDI-TOF MS method for quantification of IGF1 from human plasma samples [158]. The limit of detection for the IGF-1 MSIA was evaluated and established to be approximately 15 pM.

An important application of protein enrichment method could be in detection and measurement of mutant proteins. Genome-wide analysis has shown that solid tumors typically contain 20–100 protein-encoding genes that are mutated [159]. A small fraction of these changes are “drivers” as cancer causing events; the remainder is “passengers,” providing no selective growth advantage [160]. These proteins could be source of unique biomarker candidates. Unlike wild type protein biomarkers the mutant proteins are produced only by tumor cells. Moreover, they are not simply associated with tumors, but in the case of driver gene mutations are directly responsible for tumor generation [161]. A large number of disease-causing mutations are missense mutations that alter the encoded proteins only subtly, the detection of which can be very complicated mainly because there are few antibodies that can reliably distinguish mutant from normal versions of proteins. Because many different mutations can occur in a single cancer-related gene, it is necessary to develop a specific antibody for each possible mutant epitope which can be costly and time-consuming. Another approach measures the activity of mutant proteins but it is not generally applicable because no activity-based assays are available for most proteins. MS has been previously used to detect and quantify somatic mutations at the DNA level but not at the protein level [162]. To address this need, Vogelstein lab [161] developed an MS approach that could identify and quantify somatically mutant proteins in a generally applicable fashion. Using Ras and its mutants (most mutations clustered at residues 12 or 13 of the protein), they immunoprecipitated the Ras protein from human colorectal cancer cell line SW480 and it was then eluted and concentrated. More than 90% of the total cellular K-Ras protein was captured successfully from the lysates and eluted from the beads. Upon digestion and inclusion of heavy isotope-labeled synthetic peptides as internal controls, SRM was performed. The list of parent and product ions that were used for SRM included those representing trypsinized normal (WT) Ras protein as well as the two most common mutants of Ras in pancreatic cancers, K-Ras G12V and G12D. The summed peak intensities for the ions corresponding to the heavy and light versions of peptides representing WT and mutant proteins showed that they were related linearly to abundance across more than two orders of magnitude (10–2,000 fmol; ^2^ *R* > 0.99 for WT and mutant proteins) [161]. Similarly, they found that mutant Ras proteins could be detected and quantified in clinical specimens such as colorectal and pancreatic tumor tissues as well as in premalignant pancreatic cyst fluids. In addition to answering basic questions about the relative levels of genetically abnormal proteins in tumors, this approach could prove useful for diagnostic applications. One potential limitation of this method is its sensitivity. Results from Vogelstein report estimated that SRM can be used to detect mutant proteins reliably when they are present at levels as low as 25 fmol/mg of total protein (~6,000 cells). However, this sensitivity may be inadequate to detect mutant proteins in some clinical samples, such as sputum, serum, or urine [161]. As indicated above while the affinity MS approaches can improve the sensitivity for quantification of low-abundance proteins or mutants, the major drawback of protein capture method is that antibodies are typically not available for newly discovered biomarker candidates. The need for different antibodies for individual proteins inherently limits the multiplexing power and the throughput for quantifying a large number of target proteins when employing affinity MS approaches.

### 5.8. Peptide Capture Enrichment

An alternative for protein enrichment is to affinity capture for target peptides using anti-peptide antibodies, in which target peptides act as surrogates for protein quantification. Anderson et al. [163] introduced this method in 2004 using immobilized anti-peptide polyclonal rabbit antibodies to capture and, subsequently, elute the target peptides of four blood plasma proteins along with isotope-labeled peptide standards for MS quantification. This strategy, termed stable isotope standards and capture by anti-peptide antibodies (SISCAPA), and recent studies suggested that more than a 1000-fold enrichment can be achieved for plasma-digested peptides [164] with low ng/mL LOQs in plasma with CVs < 20% [141]. If stable isotope-labeled recombinant protein standard is available, it can be added to the biofluid in the beginning of the assay workflow to control for proteolytic efficiency. Upon digestion of the biospecimens and addition of known amounts of SIS, both spike-in and endogenous peptides are specifically enriched and their relative amounts are quantitated by MRM-MS. MS detector provides quantitation through peak areas for targeted* m/z* values. The SISCAPA strategy has been further optimized using a magnetic-bead-based platform, which can be performed in an automated fashion using 96-well plates [164, 165]. This strategy was implemented in a nine-target peptide multiplexed SISCAPA assay in which more sensitive detection in the 50–100 pg/mL range of protein concentration was reported when plasma volume was increased from 10 *μ*L to 1 mL for SISCAPA enrichment [166].

One advantage of mAbs over polyclonal antibodies in SISCAPA assays is their higher specificity and as such Schoenherr et al. demonstrated that mAbs can be configured in SISCAPA assays and reported a platform for automated screening of mAbs [167]. In another more recent report, MALDI immunoscreening (MiSCREEN) was developed, enabling rapid screening and selection of high affinity anti-peptide mAbs [168]. In other studies immuno-MALDI (iMALDI) was used where affinity-bound peptides on a MALDI utilize MALDI matrix solvents to elute the bound peptides from the beads and the resultant peak height or peak area of the peptide from an MS spectrum is used for quantitation [169, 170]. While iMALDI can be performed with only a MALDI-MS instrument, it can also be used in the MRM mode on a MALDI-MS/MS instrument (iMALDI^+^) [171].

Despite sensitivity, multiplexing, and throughput, some limitations of SISCAPA include a relatively high cost of Ab generation; long lead time (~24 weeks for assay generation) for SRM assay development [172]; success rate for producing potent antibodies as well as the potentially low peptide capture rate [166]; and background bead nonspecific binding.

The SISCAPA strategy has been also deployed in a clinical setting where the potential of the SISCAPA-SRM assays was illustrated by Hoofnagle et al., who implemented SISCAPA assays for quantification of low-abundance serum thyroglobulin, and simultaneous measurement of apolipoprotein A–I and apolipoprotein B [133, 173].

In collaboration with the Fred Hutchinson Cancer Research Center, Bio-Rad Laboratories has developed a SISCAPA training/QC kit which is currently in beta testing. The kit enables researchers who are new to the SISCAPA technique to implement an assay in their lab and gain experience with the process. Based on an assay for a murine osteopontin (OPN) peptide in a human plasma matrix, the kit provides researchers with the reagents and information needed for carrying out an assay, including a detailed standard operating procedure, antibody, heavy and light peptides, magnetic beads, and other necessary buffers and reagents. Results will be comparable to expected values, making the kit a valuable resource for quality control of the SISCAPA process.

### 5.9. MRM Reference Libraries

Access to reference spectral fragmentation libraries of proteotypic peptides and chromatographic retention time would be extremely useful for the generation of maximal product ion signal and the proteomics community in MRM-MS assay development. Skyline [174] and MRMer [175] are two open source software for developing MRM-MS-based assays by the proteomics community. Skyline (downloadable from https://brendanx-uw1.gs.washington.edu/labkey/project/home/software/Skyline/begin.view) can be integrated with all major instrument platforms and has been used to design MRM-MS assays and support data analysis including SIS. Skyline supports all major publicly available spectral libraries such as the GPM, National Institute of Standards and Technology (NIST), and the Institute for Systems Biology. Library files from these sources can be downloaded and searched with the Skyline Spectral Library Explorer, to help choose peptide precursor and product ions to monitor specific proteins of interest [174]. MRMer was developed for organizing highly complex MRM-MS experiments, including quantitative analyses using heavy/light isotopic peptide pairs, and has the capability of importing data in a platform-independent mzXML format. MRMer extracts and infers precursor-product ion transition pairings, computes integrated ion intensities, and permits rapid visual curation for analyses exceeding 1000 precursor-product pairs [175].

Automated and targeted analysis with quantitative SRM (ATAQS) [176] is another open source software which supports MRM assay development (http://tools.proteomecenter.org/ATAQS/ATAQS.html). ATAQS is an integrated software platform that supports all stages of targeted, SRM-based proteomics experiments including target selection, transition optimization, and postacquisition data analysis. This software has the potential to significantly facilitate the use of targeted proteomic techniques and contribute to the generation of highly sensitive, reproducible, and complete datasets that are particularly critical for the discovery and validation of targets in hypothesis-driven studies in systems biology. ATAQS also provides software API (application program interface) documentation that enables the addition of new algorithms to each of the workflow steps [176]. mProphet is another fully automated system that computes accurate error rates for the identification of targeted peptides in SRM data sets and maximizes specificity and sensitivity by combining relevant features in the data into a statistical model [177].

Another important source of targeted proteomic assays is SRMAtlas, which enables detection and quantification of proteins in complex proteomes (http://www.mrmatlas.org/) [178]. The information in this database results from MRM-MS measurements of natural and synthetic peptides performed on a QQQ-MS. Currently, this database allows users to query transitions from peptides from yeast, human, and mouse species obtained from QQQ-MS instruments, supplemented with ion trap (IT) observations and predictions where QQQ spectra are unavailable. An algorithm called automated detection of inaccurate and imprecise transitions (AuDIT) has been developed that can assist in MRM by automatically identifying inaccurate transition data based on the observation of interfering signal or inconsistent recovery among replicates [179]. This algorithm evaluates MRM-MS data by comparing the relative product ion intensities of the analyte peptide to those of the SIS peptides, followed by a* t*-test to determine any significant difference. The algorithm has already demonstrated the capability of identifying problematic transitions and achieving accuracies of 94–100% for the correct identification of errant transitions [179].

### 5.10. Assay Portal Community Resource

While many MRM-based assays have been published, the information is dispersed throughout the literature, and protocols for characterization of assay performances have not been standardized. Furthermore the use of MRM and its potential utility have not been realized by the biological and clinical research communities. To address these issues, the Clinical Proteomic Tumor Analysis Consortium (CPTAC 2) of the NCI has launched an assay portal to serve as a public resource of well-characterized quantitative mass spectrometry-based proteomic assays with associated SOPs, reagents, and assay validation data (http://assays.cancer.gov/). The portal database is tied to Panorama, an open source platform allowing for efficient collection and sharing of data in a vendor-neutral format. SOPs describing sample preparations are also available for download for each assay. Data quality is a major emphasis of the portal. Guidelines for MRM assay “fit-for-purpose” validation have also been established within the NCI-funded CPTAC 2, with input from the outside community solicited via a workshop [138]. To ensure uniform presentation and adequate data for establishing the accepted performance of the assays, a guidance document describing the minimal characterization data required for assay inclusion in the CPTAC assay portal has been made available for download on the portal. Five experiments of assay validation are described. Experiments 1 and 2 contain information on the assay sensitivity and linear range (determined through a response curve) and the repeatability (determined by analyzing validation samples on multiple days) and are the minimal validation requirement to qualify for inclusion in the CPTAC assay portal. A higher level of validation contains additional experiments to measure the selectivity, stability, and detection of endogenous analyte. At the time of launch, the portal contains ~462 assays with characterization data and SOPs. The CPTAC program will add several hundred more assays over the next 2-3 years, and, in the near future, the portal will be open for contributions from the community at large. The portal is able to accept data from any targeted mass spectrometry-based quantification method.

## 6. Proteomic Reproducibility and Standardization

Resistance about the validity of proteomics analyses persisted for several years in the late 90s and early 2000s. This stemmed partly from some questionable work in the pioneering years of large-scale protein identification. Many of the early landmark papers in the last 10–15 years were obtained on low-resolution instruments and without proper statistical analysis [44]. It was later found that a large proportion of the identifications obtained from such studies were false positives [180]. Many of the early problems were ascribed to the use of SELDI resulting from poor analytical technique with significant reproducibility issues. Some high profile papers were later shown to be invalid, which tainted the whole field for a while. For example a method for early ovarian cancer diagnosis was reported by a group of outstanding investigators that used mass spectrometry to detect proteomic patterns from serum samples in 2002. The reported sensitivities and specificities were approximately 100%, even for serum from patients with early-stage ovarian cancer [181]. This paper has received a few thousand citations since its publications [182]. The combination of quality investigators, the high profile journal that published the data, and a powerful editorial generated widespread media coverage and euphoria that a new era in cancer diagnostics has started. However, reports of methodological shortcomings of this method and questions about its validity were published soon after its publication [183–185]. Subsequently, others have identified bioinformatic artifacts and issues regarding variations in sample collection and storage that compromised the conclusions of the paper [184]. Despite positive publications that used similar approaches for other cancer types [186, 187], an independent validation study of this method for prostate cancer diagnosis, sponsored by Early Detection Research Network, has shown that the method does not reliably detect prostate cancer [188].

The issue of reproducibility was further exacerbated when Bergeron and colleagues published a study in 2009 [189]. The researchers sent standardized samples containing 20 known proteins to 27 labs for proteomics analysis. Each protein contained one or more unique tryptic peptides, which should have shown up in MS analysis. Only 7 out of the 27 labs initially reported all 20 proteins correctly, and only one saw all the proteotypic peptides. Yet centralized analysis of the raw data revealed that all 20 proteins and most of the peptides had been detected in all 27 labs. The message of this study was that, irrespective of instrumental method, the technology delivers high quality MS data. In contrast, this centralized analysis determined missed identifications (false negatives), environmental contamination, database matching, and curation of protein identifications as sources of problems. One suggestion was that improved search engines and databases were needed for mass spectrometry-based proteomics [189].

In a separate study, the proteomics research group of the Association of Biomolecular Resource Facilities (ABRF) sponsored a number of research studies designed to enable participants to try new techniques and assess their capabilities relative to other laboratories analyzing the same samples. This study was designed to explore the use of different approaches for determining quantitative differences for several target proteins in human plasma that were centrally prepared. These results provide a cross-sectional view of current methodologies as well as a vehicle for sharing information regarding experimental protocols and education for the proteomics community and highlight that establishing good laboratory practices is important [190]. ABRF effort to assess individual laboratory's platforms, methods, and results was one of the first attempts to address variability in sample preparation and processing on different proteomic platforms.

To address many of the critical challenges in proteomics including the lack of an ability to accurately and reproducibly measure a meaningful number of proteins in biospecimens across laboratories, the National Cancer Institute (NCI) launched the Clinical Proteomic Technologies for Cancer (CPTC) in 2006 (http://proteomics.cancer.gov/). The overall goals of CPTC were focused on removing several of the major barriers in proteomics research to enable the accurate, efficient, and reproducible identification and quantification of meaningful numbers of proteins that could drive high value protein quantitation and qualification studies. Achieving this goal would provide a firm foundation for the field of discovery proteomics and enable the rational development of clinical biomarkers to address various needs in cancer drug development, diagnostics, and clinical management. CPTC took a multidisciplinary, multi-institutional approach through its CPTAC 1 network in addressing the long-standing problems of variability issues in proteomics resulting in large measurement noise from analytical platforms rather than assessing real biological differences. CPTAC 1 carried out the first quantitative assessment of discovery proteomics technology platforms across laboratories defining a set of performance standards for identifying the sources of variability [191–193], developed a standard yeast proteome reference material available to the community through NIST for investigators to benchmark their own performance [191, 194], and developed a quality control tool to monitor and troubleshoot instrument performance (http://peptide.nist.gov/software/nist_msqc_pipeline/NIST_MSQC_Pipeline.html). The yeast protein extract (RM8323) developed by NIST under the auspices of NCI's CPTC initiative is currently available to the public (https://www-s.nist.gov/srmors/view_detail.cfm?srm=8323) and offers researchers a unique biological reference material. RM8323 is the most extensively characterized complex biological proteome and the only one associated with several large-scale studies to estimate protein abundance across a wide concentration range. The yeast protein extract and its associated reference datasets [191, 194] can be used by the research community for benchmarking instrument detection efficiency for analysis of complex biological proteomes, to improve upon current methods and to evaluate new platforms when developed.

To address the issue of reproducibility in targeted proteomics, CPTAC 1 also spearheaded a multi-institutional study composed of three substudies designed to increase the level of complexity in sample preparation at eight individual sites [135]. Intralaboratory variability and reproducibility in all three substudies were evaluated by comparing the measured concentrations of seven target proteins (human C-reactive protein, PSA, aprotinin, leptin, myoglobin, myelin basic protein, and peroxidase) to the actual concentrations across the range of spiked-in analytes (a total of nine concentration points with LOQ at 2.92 nM, that is, 73.3 ng/mL for C-reactive protein) and by determining the CVs for these quantitative measurements. The results showed that the reproducibility and precision of these quantitative measurements for nine of ten peptides tested across eight laboratories ranged from 4 to 14%, 4 to 13%, and 10 to 23% interlaboratory CVs at or near the estimated LOQ of 2.92 nM for studies I, II, and III, respectively. Intralaboratory CVs were usually <15% and <25% at the identical concentration for studies I, II, and III. The incremental increases in CVs indicate that sample handling contributes more to assay variability than instrumental variability. Robotic automation of sample handling can furthermore improve analytical variability. Very recently, CPTAC 1 teams developed a system suitability protocol (SSP), which employs a predigested mixture of six proteins, to facilitate performance evaluation of LC-SID-MRM-MS instrument platforms, configured with nanoflow-LC systems interfaced to triple quadrupole mass spectrometers [195]. The SSP was designed for use with low multiplex analyses as well as high multiplex approaches when software-driven scheduling of data acquisition is required. Performance was assessed by monitoring of a range of chromatographic and mass spectrometric metrics including peak width, chromatographic resolution, peak capacity, and the variability in peak area and analyte retention time (RT) stability. The SSP, which was evaluated in 11 laboratories on a total of 15 different instruments, enabled early diagnoses of LC and MS anomalies that indicated suboptimal LC-MRM-MS performance. Robust LC-SID-MRM-MS-based assays that can be replicated across laboratories and ultimately in clinical laboratory settings require standardized protocols to demonstrate that the analysis platforms are performing adequately and therefore use of a SSP helps to ensure that analyte quantification measurements can be replicated with good precision within and across multiple laboratories and should facilitate more widespread use of MRM-MS technology by the basic biomedical and clinical laboratory research communities.

## 7. Clearance of MS-Based Platforms for Clinical Use 

As indicated previously a typical protein biomarker discovery pipeline has three phases: discovery, verification, and clinical validation. The discovery work often uses research-grade samples from underpowered cohorts with limited sample numbers. Promising candidates from the discovery phase are verified by analyzing their performance in medium-sized clinical cohorts (*n* > 100) using standardized analytical platforms. Assays are then developed to validate the top performing candidates in larger clinical cohorts (*n* > 200). Ultimately, the utility of the validated candidates for routine clinical use is demonstrated in a clinical setting (improvement over gold standard diagnosis, cost, clinical outcomes, etc.) [196]. The path from development of biomarkers to clinical practices could take many possible steps. However, it is unequivocal that, prior to clinical use, any biomarkers have to prove their safety and efficacy in independent clinical trials using an appropriate study population for a clearly defined intended use. Three issues that are the key links in the path from discovered candidates to actual clinical diagnostics include generation of sufficient and portable evidence in preliminary validation studies; defining clinical utility for regulatory approval; and selecting/developing assays with analytical performance suitable for clinical deployment [197].

While proteomic methods may not yet be ready for implementation in routine clinical chemistry laboratories, the goal seems attainable in the near future [20]. Currently, LC-MS instruments are widely used in clinical laboratories for diagnosis of inborn errors of metabolism or for therapeutic drug monitoring and toxicology. MALDI-TOF-MS profiling methods have been recently implemented very successfully in clinical microbiology laboratories for simpler samples such as microbial colonies for identification of microorganisms, thus proving the validity of the approach [20]. BioMérieux recently announced US FDA clearance for VITEK MS, an evolutionary technology which reduces microbial identification from days to minutes (http://www.biomerieux-usa.com). VITEK MS is the first clinical mass spectrometry MALDI-TOF-based system available in the US for rapid identification of disease-causing bacteria and yeast. VITEK MS accuracy was compared to 16S ribosomal RNA gene sequencing, the gold standard, for a number of microbial categories. The overall accuracy of VITEK MS compared to nucleic acid sequencing for these organisms was 93.6 percent.

In addition, Bruker Corporation recently announced that it has been granted US FDA clearance under section 510(k) to market its MALDI Biotyper CA System in the United States for the identification of Gram negative bacterial colonies cultured from human specimens (http://www.bruker.com/). The* MALDI Biotyper* enables molecular identification and taxonomical classification or dereplication of microorganisms like bacteria, yeasts, and fungi. This is achieved using proteomic fingerprinting by high throughput MALDI-TOF mass spectrometry. The* MALDI Biotyper* uses specific proteomic fingerprints from bacterial strains. However, human protein analysis represents a level of complexity over drugs or bacterial proteins, thereby imposing particular constraints.

Another proteomic test that has been recently cleared by FDA is OVA1. OVA1 test is an* in vitro* Diagnostic Multivariate Index Assay (IVDMIA) for assessing ovarian cancer risk in women diagnosed with ovarian tumor prior to a planned surgery. OVA1 analyzes 5 proteomic biomarkers in serum and the results are combined through an algorithm to yield a single-valued index within the range of 0–10. OVA1 provides additional information to assist in identifying patients for referral to a gynecologic oncologist. In a prospective multiple-center clinical study, the addition of OVA1 in preoperative clinical assessment was found to improve sensitivity in the prediction of malignancy for ovarian tumor. OVA1 is intended to assess preoperatively the risk of ovarian cancer in women scheduled for surgery due to suspected ovarian cancer. The test result aids in the decision to refer the patient to a gynecologic oncologist for surgery for better long-term outcome [197].

To empower the scientific community with the right tools and to serve as a preview of the regulatory mindset and direction for multiplex protein assays, NCI's CPTAC 1 submitted 2 protein-based multiplex assay descriptions to the Office of* In Vitro* Diagnostic Device Evaluation and Safety of the FDA. The objective was to evaluate the analytical measurement criteria and studies needed to validate protein-based multiplex assays. Each submission described a different protein-based platform: a multiplex immunoaffinity mass spectrometry platform for protein quantification and an immunological array platform quantifying glycoprotein isoforms. Submissions provided a mutually beneficial way for members of the proteomics and regulatory communities to identify the analytical issues that the field should address when developing protein-based multiplex clinical assays. The goal of these submissions was to demonstrate the process and interactions between the sponsor and the FDA in a fashion similar to how they would proceed generally. Additionally, the feedback provided by the FDA generates some insight into the review issues that are relevant to these types of tests. Because the sponsors of the 2 mock submissions did not submit full responses and appropriate data, and because they submitted hypothetical data in large part, many issues and problems were not mentioned and discussed. For these reasons, this document is not meant to be inclusive of all the requirements for any future submission that would be made to the FDA [198].

To propel the adaptation of proteomics in clinical chemistry, important developments in workflows and instrumentation are necessary before various proteomic methods can compete with protein immunoassays performed on high throughput immunoanalyzers. The best chance for short-term application of proteomic methods in clinical chemistry laboratories will most likely be to capitalize on specific aspects of proteomic analysis such as targeting new types of biomarkers [199, 200] and offering new diagnostic solutions for orphan clinical problems [201]. In this context, SRM appears as potential alternative to classical immunoassays by combining analytical specificity and reliable quantification as described before. Among the advantages of SRM methods over classical immunoassays is the possibility of applying multiple protein tests on a single instrument without relying on commercial reagents. SRM thus can offer opportunities for measuring biomarkers in specific clinical areas that do not represent large markets for diagnostic industries. It might also be a way to reduce reagent costs such as antibodies borne by clinical laboratories. Therefore, the project for developing reliable SRM assays for a large set of human proteins is clearly of great interest [202]. It could promote broader access to this technology and, in turn, greatly facilitate application to clinical studies and increased use within clinical chemistry laboratories.

## 8. Proteogenomics

Proteogenomics, the integration of proteomic and genomic data, has recently emerged as a significant area of activity in proteomics as a promising potential approach to Omics research. The notion is based on the premise that protein data can shed light on the consequence of various genomic features, allowing researchers to determine, for example, whether or not a specific genetic variant may actually become a functional protein. Independently or as part of large-scale initiatives, a number of researchers are pursuing such studies including CPTAC 2, TCGA, and the National Human Genome Research Institute's Encyclopedia of DNA Elements Consortium (ENCODE). To a large degree, this surge in activity on the proteogenomics studies originates from advances within proteomics that have made it feasible to obtain coverage comparable to that achieved by genomics and transcriptomics. A requirement for good systems biology studies is the need to have good enough coverage in proteomics. In the last few years, obtaining coverage in the range of 10,000 to 12,000 proteins has become routine for some labs [203, 204]. In their recent work, for instance, Lehtiö and his colleagues identified 13,078 human and 10,637 mouse proteins including 39,941 peptides not previously present in the Peptide Atlas' human dataset. They also identified 224 novel human and 122 novel mouse peptides, which mapped to 164 and 101 genomic loci, respectively [205]. Using high resolution isoelectric focusing (HiRIEF) at the peptide level in the 3.7–5.0 pH range and accurate peptide isoelectric point (pI) prediction, Lehtiö probed the six-reading-frame translation of the human and mouse genomes and identified 98 and 52 previously undiscovered protein-coding loci, respectively [205].

In a separate study, Heck and his colleagues have completed a proteogenomics study of rat liver tissue, integrating whole genome sequencing, RNA-seq, and mass spec-based proteomics [206]. In this study the researchers identified 13,088 proteins, making it one of the most comprehensive proteome analyses performed to date. Integrating their genomics data, they were also able to validate 1,195 gene predictions, 83 splice events, 120 proteins with nonsynonymous variants, and 20 protein isoforms with nonsynonymous RNA editing. The effort also provided several biological insights such as the question of RNA editing—a process in which modifications are made to the sequence of an RNA molecule after it has been generated. While they indicate that RNA editing may occur, they may not often lead to a stable protein [206]. Enhancing mass spec sequence coverage as well as robust data analysis pipeline may further improve our protein variant detection.

Leveraging large-scale cancer genomics datasets, NCI is leveraging its investment in genomics through CPTAC 2, which is applying proteomic technologies to systematically identify proteins from genomically characterized tumors, such as those from TCGA program. The goal of the CPTAC 2 program is to illuminate the complex proteogenomic relationship between genomic (DNA, RNA) and proteomic (protein) abnormalities, thus producing a deeper understanding of cancer biology. The CPTAC 2 program is analyzing more than 300 samples from colorectal, breast, and ovarian cancer. A key component of the consortium is developing novel methods to integrate and visualize proteomic and genomic data to better comprehend the biological processes of the cell. The integration of terabytes of genomic and proteomic data is catalyzing the development of new computational tools and also leveraging findings from other fields such as machine learning and computer science to better understand biological processes. After this analysis, proteins of interest are selected as targets for highly reproducible and transportable assays. All of the data and resulting assays are made publicly available to help advance research in cancer biology and improve patient care. The first set of proteogenomic data were released in September 2013 (http://proteomics.cancer.gov/).

Technological advances in both the proteomics and genomics now provide the ability to discriminate genetic and posttranscriptional polymorphisms at the proteome level. The synergistic use of genomic, transcriptomic, and proteomic technologies significantly improves the data that can be gained from proteomics as well as genomics efforts. By matching deep MS-based proteomics to a personalized database built from a sample-specific genome and transcriptome, thousands of peptides that would otherwise escape identification can be identified. Such powerful tools and data demonstrate the power of and need for integrative proteogenomic analysis for understanding genetic control of molecular dynamics and phenotypic diversity in a system-wide manner which appears to be the future direction [206].

## 9. Concluding Remarks

The acceleration of biological knowledge through the mapping of HGP has resulted in the development of new high throughput next-generation sequencing (NGS) techniques. NGS analyses such as whole genome sequencing (WGS) and total RNA sequencing (RNA-seq) cannot however predict with high confidence the effects on the proteins and their variations including composition, function, and expression levels. Therefore, the completion of the HGP has also presented the new challenge of human proteome characterization using MS-based proteomics. Each of these technologies is capable of comprehensive measurements of gene products at a system level [207, 208]. Although MS and NGS are highly complementary, they have not yet effectively been integrated in large-scale studies [209] and sparsely performed in organisms with smaller genomes [210]. To correctly delineate the effects of genomic and transcriptomic variation on molecular processes and cellular functioning, integrative analyses of different data types, ideally derived from the same samples, are required [211]. The integration and interrogation of the proteomic and genomic data will provide potential biomarker candidates which will be prioritized for downstream targeted proteomic analysis. These biomarker targets will be used to create multiplex, quantitative assays for verification and prescreening to test the relevance of the targets in clinically relevant and unbiased samples. The outcomes from this approach will provide the community with verified biomarkers which could be used for clinical qualification studies; high quality and publicly accessible datasets; and analytically validated, multiplex, quantitative protein/peptide assays and their associated high quality reagents for the research and clinical community.

State-of-the-art MS approaches can routinely identify over 10,000 proteins in a single experiment [203, 204] which suggests that the analysis of complete proteomes is within reach [212]. The comprehensive human proteome project however still faces challenges including very large number of proteins with PTMs, mutations, splice variants, the variety of technology platforms, and sensitivity limitations in detecting proteins and aberrations present in low abundances. Future proteomic undertakings should continue to support technology development, optimization, and standardization. Incorporation of the most up-to-date and efficient technologies is critical in successfully advancing the translation of proteomic findings into clinically relevant biomarkers. Meanwhile, rigorous assessment of biospecimen and data quality through quality assessment criteria at each step of the biomarker development pipeline should continue to be supported. These efforts, combined with continued collaborations with regulatory agencies and clinical chemists, will expedite the development of individualized patient care through clinical proteomics.

## Abbreviations

SRM-MS: Selected reaction monitoring-MS
MRM-MS: Multiple reaction monitoring-MS
TCGA: The Cancer Genome Atlas
CPTC: Clinical Proteomic Technologies for Cancer
CPTAC 1: Clinical Proteomic Technology Assessment for Cancer
CPTAC 2: Clinical Proteomic Tumor Analysis Consortium
PTMs: Posttranslational modifications
ESI-LC-MS: Electrospray ionization-liquid chromatography tandem mass spectroscopy
MALDI-TOF: Matrix assisted laser desorption ionization time of flight
SELDI-TOF: Surface enhanced laser desorption ionization time of flight
MALDI-MSI: MALDI MS imaging
2-DE: Two-dimensional gel electrophoresis
LCM-MS: Laser capture microdissection-MS
SILAC: Stable isotope labeling with amino acids in cell culture
SISCAPA: Stable isotope standards and capture by anti-peptide antibodies
PSAQ: Protein standard absolute quantification
SID: Stable isotope dilution
SIS: Stable isotope-labelled internal standard
QQQ-MS: Triple quadrupole mass spectrometer
HGP: Human genome project
FDA: Food and Drug Administration
PRM: Parallel reaction monitoring.
